# Supplementary material for: Epigenetic Mechanisms and Nephrotic Syndrome: A Systematic Review
Source: Biomedicines. 2023 Feb 10;11(2):514. doi: 10.3390/biomedicines11020514 (PMC9953384; doi:10.3390/biomedicines11020514)
Supplement: Supplementary file 1 [file biomedicines-11-00514-s001.zip › biomedicines-2190329-supplementary.pdf]

Supplementary Table S1. Summary of the lower quality micro-RNA studies.

Publication details and reference	Study population	Epigenetic data: mechanism studied and coverage	Results: Key findings	Repeated epigenetic measures	Replication	JBI percentage and risk of bias
**Micro-RNAs - blood**						
Sui, W et al. Iranian journal of Kidney Diseases, 2014. [45]	Aged > 18 years<br><br>MPGN 10;<br>MCD 10;<br>Membranous 10;<br>FSGS 10;<br>Healthy controls 16.	Micro-RNA – blood<br><br>MiR-181a, miR-483-5p, and miR-557 primer assays.	MiR-181a was significantly upregulated in patients with MCD, MPGN, Membranous and FSGS compared to healthy controls (fold change 19.17, 20.09, 20.20 and 32.92 respectively, all $p < 0.05$).<br><br>There was no difference in miR-483-5p and miR-557 between people with NS and controls.	No	No	60%<br>Moderate risk
Teng, J et al. International Journal of Clinical & Experimental Pathology, 2015. [46]	No specific age restrictions.<br><br>NS 25;<br>Healthy controls 20.	Micro-RNA – blood<br><br>TaqMan Low density Array - 754 human miRNAs.	Participants with NS had higher expression of 35 miRNAs and lower expression of 24 miRNAs. Six of these miRNAs were confirmed with real-time PCR ($p<0.05$) and had fold changes ⩾ 1.5: miR-181a, miR-210, miR-30a, miR-942, miR-192 and miR-586.	No	No	20%<br>High risk
Zapata-Benavides, P et al. BioMed Research International, 2017. [47]	No specific age restrictions, only children included.<br><br>NS 21;<br>Healthy controls 10.	Micro-RNA – serum<br><br>MiR-15a, miR-16-1, and miR-193a primer assays.	MiR-16-1 was downregulated in NS compared to healthy controls (relative expression 0.54 compared to 1.86, $p=0.019$).	No	No	60%<br>Moderate risk
**Micro-RNAs – urine**						
Huang, Z et al. BioMed Research International, 2017. [48]	Aged < 18 years<br><br>MCD 5;<br>FSGS 8.	Micro-RNA – urinary exosomes<br><br>MiR-193a primer assay	MiR-193a levels were significantly higher in FSGS compared to MCD (1376 versus 386.3, $p <0.05$).	No	No	60%<br>Moderate risk
Wang, L et al. Frontiers in Cell and Developmental Biology, 2021. [49]	No specific age restrictions, only children included.<br><br>FSGS (non-monogenic) 8;<br>IgAN 7;<br>MCD 5;<br>Healthy controls 5.	Micro-RNA – urinary exosomes<br><br>MiR-193a primer assay	MiR-193a levels were significantly higher in FSGS compared to people with IgAN, MCD and healthy controls (1060.4, 423.7, 381.9 and 163.6 respectively, all $p < 0.05$).<br><br>For people with FSGS, miR-193a levels positively correlated with glomerulosclerosis index ($R^2 = 0.795$, $p < 0.05$).	No	No	50%<br>Moderate risk
Konta, T et al. Clinical & Experimental Nephrology, 2014. [50]	Aged > 18 years<br><br>MCD 5;<br>IgAN 46;<br>CrGN 9;<br>DN 11;<br>Healthy controls 5.	Micro-RNA – urine<br><br>Agilent human miRNA array 16.0 - 1257 human miRNAs.	The concentration of total urinary micro-RNAs were increased in patients with renal disease compared to healthy controls (median values mg/gCr: 3.6 in the healthy controls, 5.5 in the MCNS patients, 6.3 in the IgAN patients ($p <0.05$), 9.1 in the CrGN patients ($p <0.05$) and 13.0 in the DMN patients ($p <0.05$). Total urinary micro-RNA concentration was correlated with proteinuria ($R^2 = 0.42$, $p < 0.01$).<br><br>The microarray analysis detected 39 micro-RNAs that were 100 times greater in renal disease groups than healthy controls. Of these, higher levels of miR-574-3p were detected in all 4 types of renal disease.	No	No	60%<br>Moderate risk
Wang, G et al. Clinica Chimica Acta, 2013. [51]	No specific age restrictions, only adults included.<br><br>MCD or FSGS 21;<br>Diabetic glomerulosclerosis 20;<br>Membranous 23;<br>Healthy controls 10.	Micro-RNA – urine<br><br>MiR-21, miR-29a, miR-29b, miR-29c, miR-122, miR-141, miR-150, miR-184, miR-192, miR-198, miR-200a, miR-200b, miR-200c, miR-205, miR-375, miR-429 and miR-638 primer assays	MiR-192 levels were higher in patients with MCD, FSGS, Membranous and healthy controls compared to diabetic glomerulosclerosis (0.88, 0.84, 0.72 and 0.27 respectively, $p = 0.017$).<br><br>MiR-200c levels were higher in MCD, FSGS and healthy controls and lower in diabetic glomerulosclerosis and membranous (1.63, 1.04, 0.43, 0.42 respectively, $p = 0.034$).<br><br>In patients with MCD or FSGS, lower miR-184, miR-192, miR-200c and miR-275 expression correlated with lower eGFR ($R^2 = 0.55$, $p = 0.009$; $R^2 = 0.51$, $p = 0.019$; $R^2 = 0.52$, $p = 0.015$; $R^2 = 0.58$, $p = 0.006$ respectively).	No	No	60%<br>Moderate risk

**Micro-RNAs – renal tissue**						
Baker, M et al. Journal of the American Society of Nephrology, 2017. [52]	No specific age restrictions - only adults included.<br><br>DN 23;<br>FSGS 19;<br>IgAN 18;<br>MPGN 19;<br>Healthy controls 14.<br><br>Validation cohort:<br>DN 19;<br>FSGS 21.	Micro-RNA - Renal - glomeruli and proximal tubules<br><br>Illumina TruSeq small RNA - whole transcriptome.	Compared with control glomeruli, DN, FSGS, IgAN, and MPGN glomeruli exhibited differential expression of 18, 12, 2, and 17 known micro-RNAs, respectively.<br><br>Compared with control or FSGS glomeruli, IgAN glomeruli exhibited downregulated expression of hsa-miR-3182.<br><br>Compared with control proximal tubules, DN, FSGS, IgAN, and MPGN proximal tubules had differential expression of 13, 14, 8, and 8 micro-RNAs, respectively, but expression of micro-RNAs did not differ significantly between the disease groups.	No	Yes	40%<br>High risk
Lu, M et al. Experimental & Molecular Pathology, 2015. [53]	No specific age restrictions - only children included.<br><br>MPGN 22;<br>MCD 8;<br>ECPGN 11;<br>FSGS 4;<br>Membranous 3;<br>Glomerular minor lesions 4;<br>Healthy controls 8.	Micro-RNA – renal.<br><br>MiR-191, miR-151-3p, miR-150, miR-30a-5p and miR-19b primer assays.	MiR-191 and miR-151-3p intrarenal levels were higher in all 6 kidney disease groups compared to healthy controls.<br><br>MiR-19b levels were higher in membranous, MPGN and ECPGN (2.5-3 fold, $p < 0.05$].<br><br>MiR-30a-5p levels were lower in FSGS and glomerular minor lesions (0.25 and 0.5, $p < 0.05$).<br><br>MiR-150 levels were lower in MPGN, MCD, FSGS and glomerular minor lesions (0.25-0.66, $p < 0.05$).	No	No	60%<br>Moderate risk
Gebeshuber, C et al. Nature Medicine, 2013. [54]	No specific age restrictions - adults and children included.<br><br>FSGS 48 (4 with pathogenic genetic variants, 4 with HIV);<br>MCD 17;<br>Membranous 10;<br>IgAN 10;<br>Healthy controls 5.	Micro-RNA – renal – glomeruli.<br><br>MiR-193a primer assay and FISH.	MiR-193a was over expressed in glomeruli from individuals with FSGS compared to disease or healthy controls ($p < 0.0001$).	No	No	60%<br>Moderate risk
Yang, X et al. International Journal of Molecular Medicine, 2015. [55]	No specific age restrictions.<br><br>FSGS 3;<br>Healthy controls 3.	Micro-RNA – renal – glomeruli.<br><br>MiR-135a and miR-135b primer assays.	MiR-135a and miR-135b levels were higher in FSGS glomeruli compared to healthy controls ($p < 0.01$).	No	No	50%<br>Moderate risk
Yang, X et al. International Journal of Molecular Medicine, 2017. [56]	No specific age restrictions.<br><br>FSGS 3;<br>Healthy controls 3.	Micro-RNA – renal – glomeruli.<br><br>MiR-135a primer assay.	Patients with FSGS had higher levels of miR-135a compared to healthy controls ($p < 0.05$).	No	No	50%<br>Moderate risk
Peng, R et al. International Journal of Molecular Sciences, 2015. [57]	No specific age restrictions.<br><br>FSGS 1;<br>Healthy control 1.	Micro-RNA – renal – glomeruli.<br><br>MiR-30a primer assay.	Expression of miR-30a was lower in FSGS glomeruli compared to healthy control ($p < 0.05$).	No	No	20%<br>High risk
Muller-Deile, J et al. Kidney International, 2017. [58]	No specific age restrictions.<br><br>FSGS 7;<br>Membranous 9;<br>MCD 6;<br>IgAN 6;<br>DN 7;<br>Healthy controls 9.	Micro-RNA – renal – glomeruli.<br><br>In situ hybridization for miR-378a-3p.	FSGS and membranous glomeruli had increased miR-378a-3p expression compared to healthy controls ($p < 0.05$).	No	No	20%<br>High risk
Wu, J et al. Journal of the American Society of Nephrology, 2014. [59]	No specific age restrictions - only adults included.<br><br>FSGS 16;<br>Healthy controls 6.	Micro-RNA – renal – glomeruli.<br><br>MiR-30a, miR-30b, miR-30c, miR-30d, and miR-30e primer assays.	MiR-30a, miR-30b, miR-30c, miR-30d, and miR-30e levels were lower in FSGS glomeruli compared to healthy controls ($p < 0.05$ for all micro-RNAs).	No	No	60%<br>Moderate risk
**Micro-RNAs – blood and urine**						
Wang, N et al. PloS ONE, 2012. [60]	No specific age restrictions, only adults included.<br><br>FSGS 16;	Micro-RNA – blood and urine.<br><br>MiR-10a and miR-30d TaqMan probe assays	Urinary miR-10a and miR-30d levels were higher in FSGS patients compared to healthy controls ($p < 0.01$).	No	No	70%<br>Low risk

	Healthy controls 16.					

Abbreviations: Area under curve, AUC; crescentic glomerulonephritis, CrGN; diabetic nephropathy, DN; endocapillary proliferative glomerulonephritis, ECPGN; focal segmental glomerulosclerosis, FSGS; human immunodeficiency virus, HIV; IgA nephropathy, IgAN; JBI, Joanna Briggs Institute; membranoproliferative glomerulonephritis MPGN; minimal change disease, MCD; nephrotic syndrome NS; peripheral blood mononuclear cells, PBMCs.

Supplementary Table S2. Summary of the lower quality DNA methylation and small RNA studies.

Publication details and reference	Study population	Epigenetic data: mechanism studied and coverage	Results: Key findings	Repeated epigenetic measures	Replication	JBI percentage and risk of bias
**DNA methylation**						
Kobayashi, Y et al.<br>BMC Research Notes, 2017. [61]	MCD patients aged < 16 years.<br><br>MCD 6;<br>Healthy controls 5.	DNAm – monocytes and naïve T helper cells.<br><br>Microarray-based integrated analysis of methylation by isoschizomers (MIAMI) - genome wide.	In monocytes, there was differing DNAm between MCD relapse and remission (210 sites), MCD relapse and healthy controls (391 sites) and MCD remission and healthy controls (286 sites).<br><br>In naïve T cells, there was differing DNAm between MCD relapse and remission (469 sites), MCD relapse and healthy controls (445 sites) and MCD remission and healthy controls (291 sites).	Yes - paired relapse and remission	No	80%<br>Low risk
Zaorska, K et al.<br>Journal of Translational Medicine, 2021. [62]	No specific age restrictions - only children included.<br><br>SRNS 53;<br>SSNS 71;<br>Healthy controls 55.	DNAm – blood.<br><br>CpG islands for SOCS3 and SOCS5 methylation primers.	SOCS3.2 promoter was 15-fold more frequently unmethylated in SRNS compared to SSNS (p<0.0001).<br><br>The best performing steroid resistance prediction model included *ABCB1* rs1922240, rs1045642 and rs2235048, *CD73* rs9444348 and rs4431401, serum creatinine and unmethylated SOCS3 promoter region, AUC = 0.78.	No	No	60%<br>Moderate risk
Kobayashi, Y et al.<br>Pediatric Nephrology, 2012. [63]	Aged < 16 years.<br><br>MCD 6 - monocytes, 4 of these - naïve T helper cells.	DNAm – monocytes and naïve T helper cells.<br><br>MIAMI - genome wide.	Three gene loci (*GATA2, PBX4,* and *NYX*) were significantly less methylated in naïve T helper cells during MCD relapse compared to remission.<br><br>There were no differences in DNAm in monocytes between MCD relapse and remission.	Yes – paired relapse and remission	No	50%<br>Moderate risk
**Histone modifications**						
Zhang, L et al.<br>American Journal of Nephrology, 2009. [64]	No specific age restrictions - only adults included.<br><br>MCD 15;<br>Healthy controls 15.	DNAm – PBMCs.<br><br>UNH Human CpG Island Microarray (12,000 sites).	There were significant differences in H3K4me3 between people with MCD and healthy controls (848 increased probes and 231 decreased probes).<br><br>There were positive correlations between mRNA and H3K4me3 levels and an inverse relationship between promoter DNAm and H3K4me3.	No	No	100%<br>Low risk
Majumder, S et al.<br>Journal of Clinical Investigation, 2018. [65]	Aged > 18 years.<br><br>FSGS 10;<br>DN 12;<br>Healthy controls 21.	DNAm – glomeruli.<br><br>H3K27me3 immunofluorescence	There was Increased H3K4me3 in FSGS and DN compared to healthy controls (p<0.01).	No	No	70%<br>Low risk
**Long non-coding RNA**						
Han, R et al.<br>Scientific Reports, 2019. [66]	Aged > 18 years.<br><br>FSGS 5;<br>Healthy controls 5.	LncRNA – tubules.<br><br>Affymetrix HTA2.0 microarrays - whole transcriptome	LOC105375913 expression was increased in tubular cells of FSGS patients compared to healthy controls (fold change > 1.5 and false discovery rate adjusted p value < 0.05).	No	Yes	90%<br>Low risk
Hu, S et al.<br>Journal of Biological Chemistry, 2018. [67]	No specific age restrictions - only adults included.<br><br>FSGS 5;<br>Healthy controls 5.	LncRNA – glomeruli.<br><br>Affymetrix HTA2.0 microarrays - whole transcriptome and in situ hydridization staining	LOC105374325 expression was increased in glomeruli of FSGS patients compared to healthy controls (p <0.05).	No	Yes	90%<br>Low risk
Salazar-Torres, F et al.<br>Biomedical Reports, 2021. [68]	No specific age restrictions - only adults included.<br><br>FSGS 5;<br>Lupus 3;<br>ANCA vasculitis 1;	LncRNA – urine.<br><br>LncRNA TUG1 primer assay	Urinary expression of long non-coding RNA taurine upregulated gene 1 was significantly lower in patients with renal disease (p=0.015), in particular FSGS (p<0.01), compared to healthy controls.	No	No	70%<br>Low risk

	Advanced global sclerosis 1; Healthy controls 11.					
Xu, J et al. Medical Science Monito, 2020. [69]	No specific age restrictions - only children included. NS 30; Healthy controls 30.	Long non-coding RNA – PBMCs. H19 primer assay	LncRNA H19 expression was lower in PBMCs of children with NS compared to healthy controls (p<0.001).	No	No	30% High risk
**Circular RNA**						
Cui, X et al. Biochemical & Biophysical Research Communications, 2020. [70]	Aged > 18 years FSGS 6; Healthy controls 6.	Circular RNA – glomeruli. CircZNF609 FISH.	There was increased expression of circZNF609 in FSGS glomeruli compared to healthy glomeruli.	No	No	60% Moderate risk
**Abbreviations:** Area under curve, AUC; diabetic nephropathy, DN; DNA methylation, DNAm; fluorescence in situ hybridization, FISH;, focal segemental glomerulosclerosis, FSGS; IgA nephropathy, IgAN; JBI, Joanna Briggs Institute Long non-coding RNA (lncRNA; minimal change disease, MCD; membranoproliferative glomerulonephritis MPGN; nephrotic syndrome, NS; peripheral blood mononuclear cells, PBMCs; steroid resistant NS, SRNS; steroid sensitive NS, SSNS.						

References

1. Sui, W.; Lin, H.; Li, H.; Yan, Q.; Chen, J.; Dai, Y. Circulating microRNAs as potential biomarkers for nephrotic syndrome. *Iran. J. Kidney Dis.* **2014**, *8*, 371–376.
2. Teng, J.; Sun, F.; Yu, P.-F.; Li, J.-X.; Yuan, D.; Chang, J.; Lin, S.-H. Differential microRNA expression in the serum of patients with nephrotic syndrome and clinical correlation analysis. *Int. J. Clin. Exp. Pathol.* **2015**, *8*, 7282–7286.
3. Zapata-Benavides, P.; Arellano-Rodríguez, M.; Bollain-Y-Goytia, J.J.; Franco-Molina, M.A.; Rangel-Ochoa, G.A.; Avalos-Díaz, E.; Herrera-Esparza, R.; Rodríguez-Padilla, C. Cytoplasmic Localization of WT1 and Decrease of miRNA-16-1 in Nephrotic Syndrome. *BioMed Res. Int.* **2017**, *2017*, 9531074. https://doi.org/10.1155/2017/9531074.
4. Huang, Z.; Zhang, Y.; Zhou, J.; Zhang, Y. Urinary Exosomal miR-193a Can Be a Potential Biomarker for the Diagnosis of Primary Focal Segmental Glomerulosclerosis in Children. *BioMed Res. Int.* **2017**, *2017*, 7298160.
5. Wang, L.; Wang, J.; Wang, Z.; Zhou, J.; Zhang, Y. Higher Urine Exosomal miR-193a Is Associated With a Higher Probability of Primary Focal Segmental Glomerulosclerosis and an Increased Risk of Poor Prognosis Among Children With Nephrotic Syndrome. *Front. Cell Dev. Biol.* **2021**, *9*, 727370. https://doi.org/10.3389/fcell.2021.727370.
6. Konta, T.; Ichikawa, K.; Suzuki, K.; Kudo, K.; Satoh, H.; Kamei, K.; Nishidate, E.; Kubota, I. A microarray analysis of urinary microRNAs in renal diseases. *Clin. Exp. Nephrol.* **2013**, *18*, 711–717. https://doi.org/10.1007/s10157-013-0906-5.
7. Wang, G.; Kwan, B.C.-H.; Lai, F.M.-M.; Chow, K.-M.; Li, P.K.-T.; Szeto, C.-C. Urinary sediment miRNA levels in adult nephrotic syndrome. *Clin. Chim. Acta* **2013**, *418*, 5–11.
8. Baker, M.A.; Davis, S.J.; Liu, P.; Pan, X.; Williams, A.M.; Iczkowski, K.A.; Gallagher, S.T.; Bishop, K.; Regner, K.R.; Liu, Y.; et al. Tissue-Specific MicroRNA Expression Patterns in Four Types of Kidney Disease. *J. Am. Soc. Nephrol.* **2017**, *28*, 2985–2992. https://doi.org/10.1681/asn.2016121280.
9. Lu, M.; Wang, C.; Yuan, Y.; Zhu, Y.; Yin, Z.; Xia, Z.; Zhang, C. Differentially expressed microRNAs in kidney biopsies from various subtypes of nephrotic children. *Exp. Mol. Pathol.* **2015**, *99*, 590–595. https://doi.org/10.1016/j.yexmp.2015.10.003.
10. Gebeshuber, C.A. ; Kornauth, C.; Dong, L.; Sierig, R.; Seibler, J.; Reiss, M.; Tauber, S.; Bilban, M.; Wang, S.; Kain, R.; et al. Focal segmental glomerulosclerosis is induced by microRNA-193a and its downregulation of WT1. *Nat. Med.* **2013**, *19*, 481–487. https://doi.org/10.1038/nm.3142.
11. Yang, X.; Wang, X.; Nie, F.; Liu, T.; Yu, X.; Wang, H.; Li, Q.; Peng, R.; Mao, Z.; Zhou, Q.; et al. miR-135 family members mediate podocyte injury through the activation of Wnt/β-catenin signaling. *Int. J. Mol. Med.* **2015**, *36*, 669–677. https://doi.org/10.3892/ijmm.2015.2259.
12. Yang, X.; Wu, D.; Du, H.; Nie, F.; Pang, X.; Xu, Y. MicroRNA-135a is involved in podocyte injury in a transient receptor potential channel 1-dependent manner. *Int. J. Mol. Med.* **2017**, *40*, 1511–1519. https://doi.org/10.3892/ijmm.2017.3152.
13. Peng, R.; Zhou, L.; Zhou, Y.; Zhao, Y.; Li, Q.; Ni, D.; Hu, Y.; Long, Y.; Liu, J.; Lyu, Z.; et al. MiR-30a Inhibits the Epithelial—Mesenchymal Transition of Podocytes through Downregulation of NFATc3. *Int. J. Mol. Sci.* **2015**, *16*, 24032–24047. https://doi.org/10.3390/ijms161024032.
14. Müller-Deile, J.; Dannenberg, J.; Schroder, P.; Lin, M.-H.; Miner, J.H.; Chen, R.; Bräsen, J.-H.; Thum, T.; Nyström, J.; Staggs, L.B.; et al. Podocytes regulate the glomerular basement membrane protein nephronectin by means of miR-378a-3p in glomerular diseases. *Kidney Int.* **2017**, *92*, 836–849. https://doi.org/10.1016/j.kint.2017.03.005.

15. Wu, J.; Zheng, C.; Fan, Y.; Zeng, C.; Chen, Z.; Qin, W.; Zhang, C.; Zhang, W.; Wang, X.; Zhu, X.; et al. Downregulation of MicroRNA-30 Facilitates Podocyte Injury and Is Prevented by Glucocorticoids. *J. Am. Soc. Nephrol.* **2014**, *25*, 92–104. https://doi.org/10.1681/asn.2012111101.
16. Wang, N.; Zhou, Y.; Jiang, L.; Li, D.; Yang, J.; Zhang, C.-Y.; Zen, K. Urinary MicroRNA-10a and MicroRNA-30d Serve as Novel, Sensitive and Specific Biomarkers for Kidney Injury. *PLoS ONE* **2012**, *7*, e51140. https://doi.org/10.1371/journal.pone.0051140.
17. Kobayashi, Y.; Aizawa, A.; Takizawa, T.; Igarashi, K.; Hatada, I.; Arakawa, H. Changes in DNA methylation in naïve T helper cells regulate the pathophysiological state in minimal-change nephrotic syndrome. *BMC Res. Notes* **2017**, *10*, 480. https://doi.org/10.1186/s13104-017-2719-1.
18. Zaorska, K.; Zawierucha, P.; Świerczewska, M.; Ostalska-Nowicka, D.; Zachwieja, J.; Nowicki, M. Prediction of steroid resistance and steroid dependence in nephrotic syndrome children. *J. Transl. Med.* **2021**, *19*, 130. https://doi.org/10.1186/s12967-021-02790-w.
19. Kobayashi, Y.; Aizawa, A.; Takizawa, T.; Yoshizawa, C.; Horiguchi, H.; Ikeuchi, Y.; Kakegawa, S.; Watanabe, T.; Maruyama, K.; Morikawa, A. DNA methylation changes between relapse and remission of minimal change nephrotic syndrome. *Pediatr. Nephrol.* **2012**, *27*, 2233–2241.
20. Zhang, L.; Dai, Y.; Peng, W.; Lu, J.; Zhang, Y.; Wang, L. Genome-Wide Analysis of Histone H3 Lysine 4 Trimethylation in Pe-ripheral Blood Mononuclear Cells of Minimal Change Nephrotic Syndrome Patients. *Am. J. Nephrol*. **2009**, *30*, 505–513.
21. Majumder, S.; Thieme, K.; Batchu, S.N.; Alghamdi, T.A.; Bowskill, B.B.; Kabir, M.G.; Liu, Y.; Advani, S.L.; White, K.E.; Geldenhuys, L.; et al. Shifts in podocyte histone H3K27me3 regulate mouse and human glomerular disease. *J. Clin. Investig*. **2017**, *128*, 483–499. https://doi.org/10.1172/jci95946.
22. Han, R.; Hu, S.; Qin, W.; Shi, J.; Zeng, C.; Bao, H.; Liu, Z. Upregulated long noncoding RNA LOC105375913 induces tubulointerstitial fibrosis in focal segmental glomerulosclerosis. *Sci. Rep.* **2019**, *9*, 716. https://doi.org/10.1038/s41598-018-36902-2.
23. Hu, S.; Han, R.; Shi, J.; Zhu, X.; Qin, W.; Zeng, C.; Bao, H.; Liu, Z. The long noncoding RNA LOC105374325 causes podocyte injury in individuals with focal segmental glomerulosclerosis. *J. Biol. Chem*. **2018**, *293*, 20227–20239.
24. Salazar-Torres, F.; Medina-Perez, M.; Melo, Z.; Mendoza-Cerpa, C.; Echavarria, R. Urinary expression of long non-coding RNA TUG1 in non-diabetic patients with glomerulonephritides. *Biomed. Rep.* **2020**, *14*, 17.
25. Xu, J.; Ge, T.; Zhou, H.; Zhang, L.; Zhao, L. Absence of Long Noncoding RNA H19 Promotes Childhood Nephrotic Syndrome through Inhibiting ADCK4 Signal. *Experiment* **2020**, *26*, e922090. https://doi.org/10.12659/msm.922090.
26. Cui, X.; Fu, J.; Luan, J.; Qi, H.; Jiao, C.; Ran, M.; Wang, D.; Hao, X.; Zhang, Y.; Kopp, J.B.; et al. CircZNF609 is involved in the pathogenesis of focal segmental glomerulo-sclerosis by sponging miR-615-5p. *Biochem. Biophys. Res. Commun.* **2020**, *531*, 341–349.
